# The immune microenvironment of uterine adenosarcomas

**DOI:** 10.1186/s13569-020-0127-0

**Published:** 2020-03-28

**Authors:** Ali Mohammed Refaat Ali, Jen-Wei Tsai, Cheuk Hong Leung, Heather Lin, Vinod Ravi, Anthony P. Conley, Alexander J. Lazar, Wei-Lien Wang, Michael J. Nathenson

**Affiliations:** 1 Department of Translational Molecular Pathology, The University of Texas MD Anderson Cancer Center, 2130 Holcombe Blvd, Life Science Plaza (LSP11.5010), Houston, TX 77030 USA; 2 Department of Biostatistics, The University of Texas MD Anderson Cancer Center, 1515 Holcombe Blvd Unit 450, Houston, TX 77030 USA; 3 Department of Sarcoma Medical Oncology, The University of Texas MD Anderson Cancer Center, 1515 Holcombe Blvd Unit 450, Houston, TX 77030 USA; 4 Department of Medical Oncology, Center for Sarcoma and Bone Oncology, Dana-Farber Cancer Institute, 450 Brookline Ave, Boston, MA 02215 USA

**Keywords:** Adenosarcoma, Microenvironment, Immune infiltrate, Immunotherapy, CD3+, CD8+

## Abstract

**Background:**

Uterine adenosarcoma (UA) is an extremely rare sarcoma subtype. There has been limited evaluation of the immune microenvironment in these tumors. The objective of this study is to examine and describe the immune infiltrate and PD-1/PD-L1 expression in UA and to correlate these changes in the tumor micro-environment with the overall survival status or the disease-free survival status (DFSS), respectively.

**Methods:**

Patients (pts) treated at our center from 1982 to 2014 with UA were identified. Fifteen cases had tumor paraffin-embedded blocks available. Immunohistochemistry studies for CD3, CD8, FOXP3, CD163, PD-1 and PD-L1 (clone 22C3) were performed. Image analysis was used to assess the density (cells/mm^2^), except in PD-L1, where the percentage of membranous staining on tumor cells was noted.

**Results:**

Immune infiltrate analysis median (range) density in cells/mm^2^ varied broadly: CD3 178 (15–802); CD8 117 (11–661); FoxP3 4.8 (0.2–82); CD163 791 (264–1861); and PD1 5 (1–65). 3 cases had rare (1%) PD-L1 tumor membranous labeling. The reports yielded that ten pts were alive, and 5 were dead. Pts who were alive had significant higher CD3 and CD8 median densities in tumors than those who were dead (p = 0.040). There was no correlation between DFSS and CD3 or CD8 median densities. Patients who had no local recurrence had significantly higher CD3 and CD8 median densities in tumors than those who had local recurrence (p = 0.040).

**Conclusions:**

In conclusion, this is the first report characterizing the presence of immune infiltrate and PD-1/PD-L1 expression in UA. CD3+ CD8+ T-cells density may be prognostic. The immune-responsiveness of UA needs to be further investigated in a larger study.

## Background

Uterine adenosarcoma is a rare biphasic uterine sarcoma, composed of both benign, but atypical, glandular epithelial and malignant mesenchymal sarcomatous components and most often occurring in adult women. Standard treatment of uterine adenosarcoma is surgical resection through hysterectomy and bilateral salpingo-oophorectomy [1–3]. Overall survival and rate of recurrence are significantly influenced by the presence of sarcomatous overgrowth (sarcomatous component of more than 25% of the tumor), as well as myometrial invasion [4–6]. Stage, resection status, age, lymphovascular invasion, tumor size, mitosis number, and lymph nodes involvement are also prognostic [2, 4, 7]. Treatment options for patients with recurrent or metastatic disease are limited.

Immunotherapy, particularly with blockade of the PD-1/PD-L1 pathway (programmed death-1/programmed death ligand-1) has proven effective in numerous malignancies, such as malignant melanoma, non-small cell lung cancer, renal cell carcinoma, and Hodgkin’s Lymphoma, though results are still limited for sarcomas [8]. Immunotherapy trials in soft tissue and bone sarcomas, such as SARC 028, have occasionally reported responses, particularly in one patient with uterine leiomyosarcoma. Unfortunately, most sarcoma patients do not respond to immunotherapy [9–11]. It is critically important to understand the tumor immunologic microenvironment, which may help determine which sarcomas could respond to immunotherapy. The immune microenvironment of uterine adenosarcoma previously poorly examined. Tumor infiltrating lymphocytes (TILs) and PD-1/PD-L1 expression have been reported in soft tissue and bone sarcomas, including some uterine leiomyosarcomas and undifferentiated uterine sarcomas [12–15]. Pre-clinical data in other sarcoma suggest that tumor TILs or PD-1/PD-L1 expression may represent an important prognostic factor in sarcomas [12, 13]. Loss of tumor cell MHC expression may represent one possible resistance mechanisms to immunotherapy in sarcoma patients [16–18]. A 2nd mechanism of resistant is the inhibitory effect of tumor infiltrating macrophages on the tumor immunologic micro-environment [19]. Undifferentiated pleomorphic sarcoma may have the most sensitive sarcoma tumor microenvironment to immunotherapy [20]. These facts have led to the increased interest in characterizing the immune microenvironment in uterine adenosarcoma, with the hope to better understand the microenvironment of these tumors and to then improve treatment and outcomes.

In this study, our objective was to examine the immune infiltrate in terms of T cells and macrophages, and PD-1/PD-L1 expression in uterine adenosarcoma and relate it to the overall survival, disease-free survival, and clinical prognostic factors.

## Materials and methods

With the IRB’s approval, a search was conducted of the Tumor registry for patients with uterine or extra-uterine adenosarcoma treated at a MD Anderson cancer treatment center from August 1982 to December 2014. One hundred and sixty-five uterine adenosarcoma patients were identified, though only fifteen cases had tumor paraffin-embedded blocks available, the latter of which represented the study population. Tissue was obtained from hysterectomy or biopsy, and samples were treatment naïve. A tissue microarray of the fifteen cases was created. Immunohistochemistry study was conducted to examine for the presence of overall T cell lymphocytes (CD3), cytotoxic T-cells (CD8), regulatory T cells (FOXP3) and histiocytes/macrophages (CD163). Additional immunohistochemistry was performed to examine for T cell expression of PD-1 (program death) and tumoral expression of PD-L1 (program death ligand). For the evaluation of PD-L1 expression, an FDA approved assay Dako PharmDx anti-PD-L1 clone 22C3 antibody was used. This assay was chosen given it wide use and overall consistency and comparability to most anti-PD-L1 antibody clones, (PMID: 31409885, 27541827). Unstained slides were prepared and immunohistochemical studies were performed using an autostainer (Leica Biosystems, Bond III Autostainer, Buffalo Grove IL) with anti-CD3 (Rabbit polyclonal, 1:100, Dako), anti-CD8 (C8/144B, Thermo Scientific, 1:25), anti-FOXP3 (206D, Biolegend, 1:50), anti-CD163(10D6, Leica Biosystems, 1:100), anti-PD-1 (EPR4877(2), Abcam, 1:250) and anti-PD-L1 (clone 22C3, Agilent Technologies, Carpinteria, CA) markers. Image analysis on the entire tissue microarray core using Aperio analytic image software (Leica Biosystems) was performed. Lymphocytic infiltrate of the above markers together with histiocytic infiltrate were quantified as density (Number/mm^2^). For PD-L1, the percentage of membranous staining on tumor cells was determined. The areas selected for the analysis were the ones mostly representative of the tumor. The clinical and pathological characteristics of each of these uterine adenosarcoma patients were collected.

The exact Wilcoxon rank sum test was used to compare the continuous variables between patient characteristics’ subgroups [21]. The correlation between two continuous factors was measured by Spearman correlation [22]. Overall survival (OS) was defined as “from diagnosis to the time of death” or “to the time of last contact.” Disease-free survival (DFS) was defined as “from time of surgery to the time of recurrence or death,” whichever was earlier, or “to the time of last contact.” Local recurrence-free survival (LRFS) was defined as “from the time of surgery to the time of local recurrence or death,” whichever was earlier, or “to the time of last contact.” Given the small numbers of events, the associations between the immune infiltrates and time-to-events outcomes were not assessed using time-to-event analysis approaches. Instead, the exact Wilcoxon rank sum test was used to compare the immune infiltrate levels between the patients who either experienced an event and who had not experienced an event by the time of the last follow-up. Two-sided p-value < 0.05 was considered statistically significant. All statistical analyses were performed in SAS 9.4.

## Results

### Clinical and pathological characteristics

Clinical and pathological characteristics of the 15 patients included in this study are described in Table 1, and include age, tumor size, follow-up, sarcomatous overgrowth, myometrial invasion, lymphovascular invasion, stage, and estrogen and progesterone receptor status. The age distribution of adenosarcoma varied from 26 to 61 years with a mean age of diagnosis of 48 years. Fourteen patients at diagnosis had a FIGO stage I where the tumor was limited to the uterine corpus. Ten patients had sarcomatous overgrowth (SO), and nine patients had myometrial invasion (MI). None of the fifteen patients had lymphovascular (LVI) invasion. None of the fifteen patients had lymph node involvement. Only four patients had tumors that were negative for ER and PR (Table 1). At the last follow-up, 10 patients were alive and 5 patients were dead; 9 patients had no recurrence and 6 patients had disease recurrence, of the disease recurrences 5 patients had local recurrence and one patient had distant recurrence (Table 1).

**Table 1:** Patients pathologic characteristics

Patient characteristics	Median (min, max)
Age of diagnosis	48 years (26, 61)
Tumor size	5.9 cm (2.5, 11)
Follow-up period	6.6 years (2.4, 10.8)

	n (%)
ER	
Negative	4 (27)
Positive	11 (73)
PR	
Negative	4 (27)
Positive	11 (73)
Myometrial invasion	
Absent	6 (40)
Present	9 (60)
Lymphovascular invasion	
Absent	15 (100)
Present	0 (0)
Sarcomatous overgrowth	
Absent	5 (33)
Present	10 (67)
Lymph node involvement	
No	15 (100)
Figo stage overall	
I	14 (93)
II/III/IV	1 (7)
Vital status	
Alive	10 (67)
Dead	5 (33)
Disease-free survival status	
Disease-free	9 (60)
Disease recurred	6 (40)
Local recurrence-free survival status	
Local recurrence-free	10 (67)
Local recurrence	5 (33)

### Immune Infiltrates

**Fig. 1:**
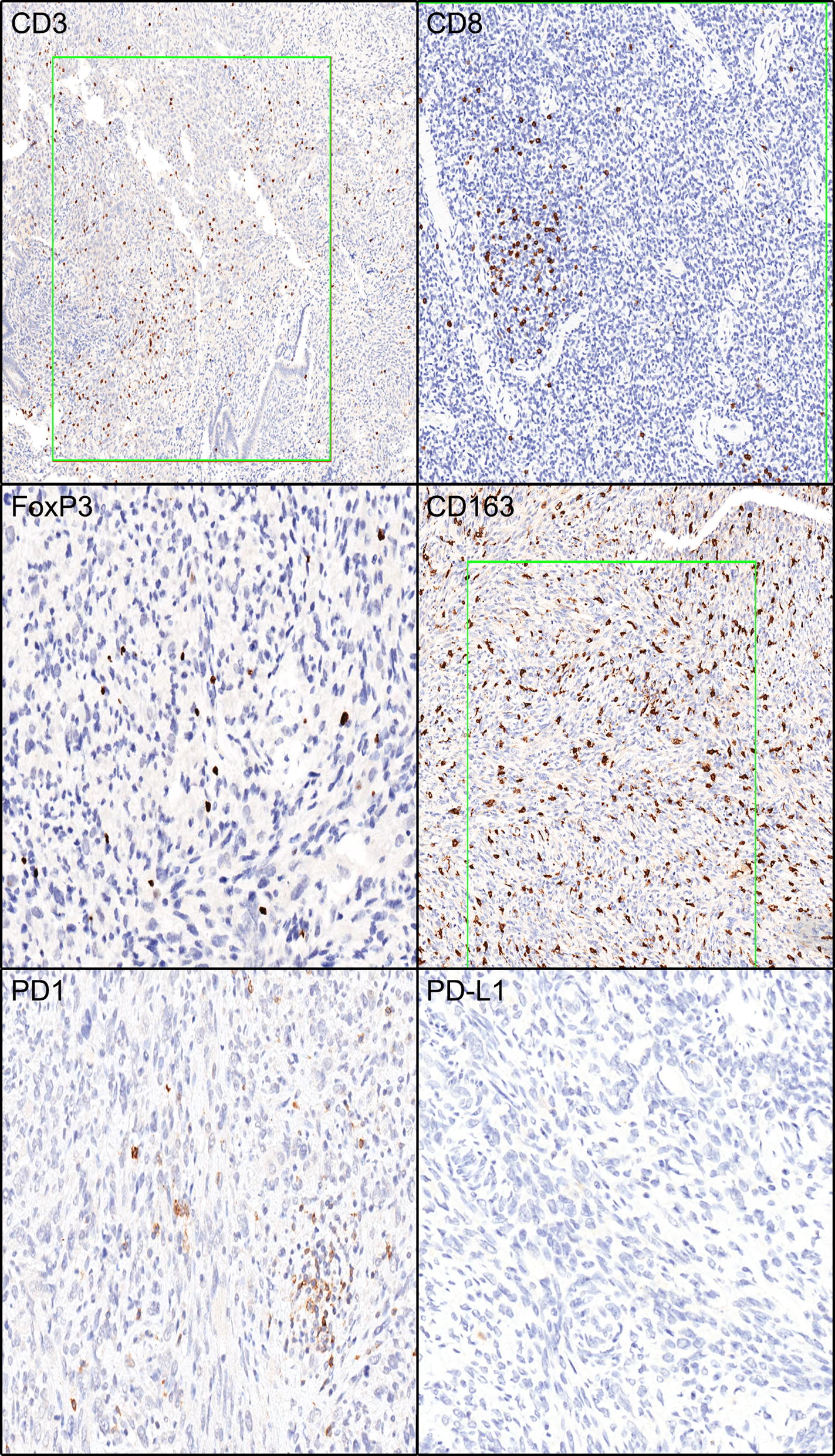
Immune infiltrate immunohistochemistry labeling. Green boxes are examples of highlighted areas used for analysis. Examples are show for IHC staining for CD3, CD8, PD-1, PD-L1, CD163, and FoxP3

The Immune infiltrate analysis median (range) density in cells/mm^2^ varied broadly for CD3, CD8, FoxP3, CD163, PD-1, and PD-L1. Table 2 shows the immune infiltrate minimum, median, maximum, mean, and standard deviation for each of these immune infiltrates. For example, the median and range for CD3 was 178 (15–802), CD8 was 117 (11–661), FoxP3 was 4.8 (0.2–82), CD163 was 791 (264–1861), and for PD1 was 5 (1–65) (Table 2). Three cases had rare (1%) PD-L1 tumor membranous labeling. Figure 1 shows examples of IHC staining for each of these immune infiltrates.

**Table 2:** Immune infiltrate characteristics

Factor	n	Min	Median	Max	Mean	SD
CD3	15	15	178	802	248.8	226.2
CD8	15	11	117	661	151.5	162
FoxP3	15	0.2	4.8	82	12.2	21.6
CD163	15	264	791	1861	918.9	547.1
PD1	15	1	5	65	11.5	16.9

The exact Wilcoxon rank sum test was used to compare the continuous variables between patient characteristics’ subgroups

### Correlation with OS, DFS, LRFS

**Fig. 2:**
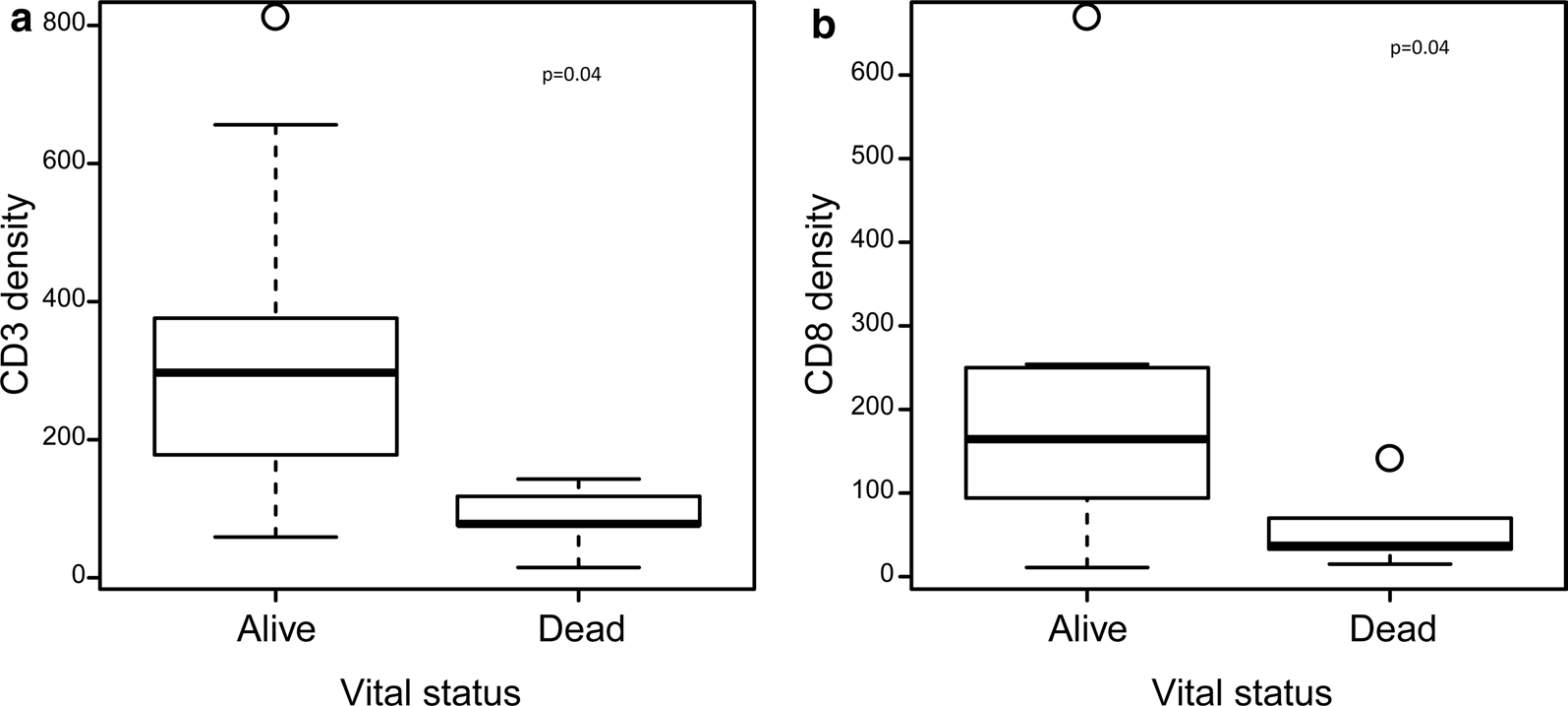
Box Plots for CD3 and CD8 Density by Overall Survival Status. Show shows a box plot comparing **a** CD3 density and **b** CD8 density to overall survival status (alive/dead)

**Fig. 3:**
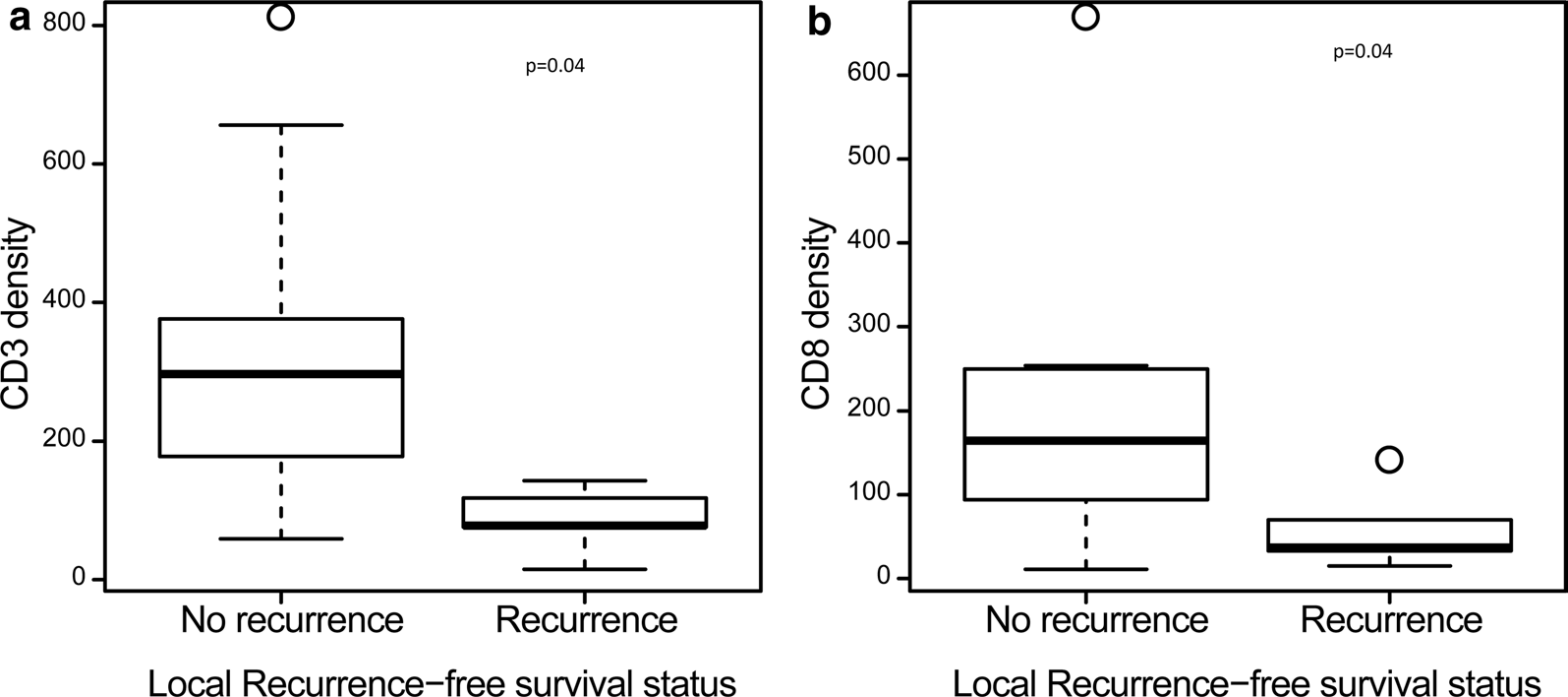
Box Plots for CD3 and CD8 Density by Local Recurrence-Free Survival Status. Show shows a box plot comparing **a** CD3 density and **b** CD8 density to local recurrence-free survival status (local recurrence yes/no)

Patients who were alive with disease, alive with NED, or censored at last follow-up had a significantly higher CD3 and CD8 median densities in their tumors than those who were dead of disease (p = 0.040). (Table 3, Fig. 2). There was no correlation between median FoxP3 and CD163 densities, or PD-1 expression and overall survival status. There was no correlation between disease-free survival status and CD3 or CD8 median densities (Table 4). There was no correlation between median FoxP3 and CD163 densities, or PD-1 expression and disease-free survival status. However, patients who were alive and had no local recurrence at the last follow-up had significantly higher CD3 and CD8 median densities in their tumors than those who were alive and had local recurrence (p = 0.040). (Table 5, Fig. 3). There was no correlation between median FoxP3 or CD163 density, or PD-1 expression and local recurrence-free survival.

**Table 3:** Comparison of the immune infiltrate by overall survival statuses

Factor	Overall, median (min, max)	Alive (n1 = 10), median (min, max)	Dead (n2 = 5), median (min, max)	p-value
CD3	178 (15, 802)	297 (59, 802)	78 (15, 143)	0.040
CD8	117 (11, 661)	165 (11, 661)	37 (15, 133)	0.040
FoxP3	5 (0, 82)	7 (0, 82)	1 (0, 14)	0.322
CD163	791 (264, 1861)	834 (399, 1861)	791 (264, 1807)	0.953
PD1	5 (1, 65)	5 (1, 65)	5 (1, 23)	0.839

The exact Wilcoxon rank sum test was used to compare the continuous variables between patient characteristics’ subgroups

**Table 4:** Comparison of the immune infiltrate by disease-free survival statuses

Factor	Overall, median (min, max)	No disease (n1 = 9), median (min, max)	Disease (n2 = 6), median (min, max)	p-value
CD3	178 (15, 802)	284 (59, 802)	98 (15, 376)	0.181
CD8	117 (11, 661)	137 (11, 661)	54 (15, 250)	0.181
FoxP3	5 (0, 82)	9 (0, 82)	2 (0, 14)	0.403
CD163	791 (264, 1861)	1090 (399, 1861)	685 (264, 1807)	0.607
PD1	5 (1, 65)	4 (1, 65)	12 (1, 26)	0.664

The exact Wilcoxon rank sum test was used to compare the continuous variables between patient characteristics’ subgroups

**Table 5:** Comparison of the immune infiltrate by local recurrence-free statuses

Factor	Overall, median (min, max)	No recurrence (n1 = 10), median (min, max)	Recurrence (n2 = 5), median (min, max)	p-value
CD3	178 (15, 802)	297 (59, 802)	78 (15, 143)	0.040
CD8	117 (11, 661)	165 (11, 661)	37 (15, 133)	0.040
FoxP3	5 (0, 82)	7 (0, 82)	1 (0, 14)	0.322
CD163	791 (264, 1861)	834 (399, 1861)	791 (264, 1807)	0.953
PD1	5 (1, 65)	5 (1, 65)	5 (1, 23)	0.839

The exact Wilcoxon rank sum test was used to compare the continuous variables between patient characteristics’ subgroups

### Correlation with prognostic factors

There was no correlation between presence or absence of myometrial invasion or sarcomatous overgrowth and median CD3 (p = 0.96, 0.31), CD8 (p = 0.86, 0.31), FoxP3 (p = 0.55, 0.082), CD163 density (p = 0.69, 0.86), or PD-1 expression (p = 0.71, 0.49), respectively. There was insufficient patients with lymph-node involvement or lymphovascular invasion or an advanced stage to correlate with the immune infiltrate. Patients with tumors negative for ER and PR had significantly higher CD163 median densities than tumors that were positive for ER and PR (p = 0.040; p = 0.040). Otherwise there was no correlation between median CD3, CD8, FoxP3 densities and PD-1 expression and estrogen and progesterone receptor status. There was no correlation between the age of the patient at diagnosis and median CD3, CD8, FoxP3, CD163 densities, or PD-1 expression.

## Discussion

To our knowledge, this study is the first of its kind and it was conducted to characterize the immune infiltrate and PD-1, PD-L1 analysis in uterine adenosarcoma.

Our study showed that patients who were (1) alive with disease, or (2) alive with no evidence of disease, had significantly higher CD3 and CD8 median densities compared to those patients who died of disease. Additionally, patients who were alive with no local recurrence had significantly higher CD3 and CD8 median densities compared to those who had a local recurrence. High levels of CD8+ T-lymphocytes have been strongly associated with prolonged overall survival and with favorable prognostic factors which, in turn, highlight the role of the immune system in endometrial cancer [23]. Similar findings have been seen in ovarian cancers where the presence of CD3 was found to be a strong prognostic factor of 10-year survival rates [24]. The presence of CD8 was associated with improved 5 and 10-year survival [24]. TILs have also been have associated with improved outcomes in some soft tissue sarcoma subtypes [13] and the presence of TILs may be a predictor of more favorable outcomes in uterine adenosarcomas.

Existing evidence suggests that the presence of TIL infiltrate in tumor tissue with an active PD-1/PDL1 expression is essential for immune checkpoint inhibitors. This evidence has been the basis for the FDA approval of certain immune therapeutic agents in the treatment of specific tumors, such as non-small cell lung cancer. The role of immunotherapy in gynecological cancers has emphasized the concepts of active immunotherapy through therapeutic vaccination, passive immunotherapy through adoptive cellular therapy using components of the immune system as antibodies and lymphocytes, and the use of immune checkpoint inhibitors which is by far the most promising yet [25]. Monoclonal antibodies against cytotoxic T lymphocytes-associated antigen 4 (CTLA-4) and PD1 and PD-L1 are currently employed in many clinical settings. Several reported and ongoing trials have looked into the role of checkpoint inhibitors in ovarian, cervical and endometrial cancers. In a trial of 20 patients involving the use of nivolumab [26], an anti-PD-1 antibody in ovarian cancer has shown a response rate of 15% and disease control rate of 45%. However, results in soft tissue sarcoma trials have been less promising. The majority of soft tissue sarcomas do not respond to immunotherapy. In fact, a trial examining immunotherapy in uterine leiomyosarcomas showed no responses.

In the present study, PD1 and PD-L1 expression was not significant in uterine adenosarcomas. Therefore, PD1-PDL1 expression may not be the best predictor of immunotherapy response in uterine adenosarcomas. The microenvironment of adenosarcoma in our cohort showed a higher density of CD8+ lymphocytes in tumor tissue in the absence of remarkable existence of PDL-1 expression by the tumor cells. The dissociation in the expression of CD8 and and PD1/PD-L1 axis suggests that adenosarcoma is a less favorable target for PD1/PD-L1 checkpoint inhibitors [27] and may use an alternate immune escape mechanism. Further investigations are required to identify immunologic therapeutic targets that may affect uterine adenosarcoma or other soft tissue sarcomas [8, 10, 28].

The most accepted prognostic factors in the literature for local adenosarcoma include the extent of myometrial invasion, sarcomatous overgrowth, lymphovascular invasion, lymph node metastasis, and age. Sarcomatous overgrowth and myometrial invasion are both associated with a more aggressive disease course, shorter relapse time, and decreased overall survival [2]. Our study did not associate the immune infiltrate median densities and the PD-1 expression with these factors. However, tumors with higher CD3 and CD8 median densities were significant determinants of overall survival. There was an association of increased macrophage infiltrate in patients with estrogen and progesterone receptor negative tumors. The significance of this finding is uncertain, even though an increased macrophage infiltrate has also been reported in triple negative breast cancer.

In our study, characterization of immune infiltrate was limited to biopsies prior to treatment. Further analysis of the immune infiltrate densities following or during treatment with radiation and systemic chemotherapy may be informative. Evaluation of HLA expression was not possible given the limited available tissue and is a further limitation of this study. Thus, the major limitation to our study is the small patient sample size, and small amount of tumor tissue, due to lack of availability of tumor blocks given the referral nature of our practice and the rarity of disease. Consequently, these results should be validated in a larger multi-center study. Despite these limitations, we were still able to identify that CD8 density may correlate with disease status. However, further studies are required to investigate the correlation of this effect with known UA risk factors, such as sarcomatous overgrowth, myometrial invasion, age, stage, and lymphovascular invasion; in an effort to improve outcomes and treatments for this incredibly rare disease. In the future, the role of immunotherapy should also be explored in the management of this rare uterine sarcoma for which there is no standard-of-care treatment in the recurrent or metastatic setting.

## Conclusions

Uterine adenosarcomas have low PD-1 and PD-L1 expression. CD3+ CD8+ T-cells density may be prognostic in uterine Adenosarcomas. The immune-microenvironment and immune-responsiveness of uterine adenosarcomas needs to be further investigated in a larger study.

## Data Availability

All data is available upon request.

## Abbreviations

UA: Uterine adenosarcoma
DFSS: Disease-free survival status
PD: 1-Programmed death-1
PD-L1: Programmed death ligand-1
TILs: Tumor infiltrating lymphocytes
MHC: Major histocompatibility complex
OS: Overall survival
DFS: Disease-free survival
LRFS: Local recurrence-free survival
SO: Sarcomatous overgrowth
MI: Myometrial invasion
LVI: Lymphovascular invasion
ER: Estrogen receptor
PR: Progesterone receptor
